# Investigating the Barriers to Applying the Internet-of-Things-Based Technologies to Construction Site Safety Management

**DOI:** 10.3390/ijerph19020868

**Published:** 2022-01-13

**Authors:** Sanaz Tabatabaee, Saeed Reza Mohandes, Rana Rabnawaz Ahmed, Amir Mahdiyar, Mehrdad Arashpour, Tarek Zayed, Syuhaida Ismail

**Affiliations:** 1 Green Cities and Construction Research Group, Razak Faculty of Technology and Informatics, Universiti Teknologi Malaysia, Kuala Lumpur 54100, Malaysia; tsanaz@utm.my (S.T.); syuhaida.kl@utm.my (S.I.); 2 Department of Building and Real Estate (BRE), Faculty of Construction and Environment (FCE), The Hong Kong Polytechnic University, Hung Hom, Kowloon, Hong Kong; saeedreza.mohandes@polyu.edu.hk (S.R.M.); tarek.zayed@polyu.edu.hk (T.Z.); 3 Department of Civil Engineering, NED University of Engineering & Technology, Karachi 75270, Pakistan; enawaz@cloud.neduet.edu.pk; 4 School of Housing, Building and Planning, Universiti Sains Malaysia, Gelugor 11800, Malaysia; 5 Department of Civil Engineering, Clayton Campus, Monash University, Melbourne, VIC 3800, Australia; mehrdad.arashpour@monash.edu

**Keywords:** digital technology, fuzzy sets, Delphi, Internet of Things, construction safety, occupational health and safety

## Abstract

The utilization of Internet-of-Things (IoT)-based technologies in the construction industry has recently grabbed the attention of numerous researchers and practitioners. Despite the improvements made to automate this industry using IoT-based technologies, there are several barriers to the further utilization of these leading-edge technologies. A review of the literature revealed that it lacks research focusing on the obstacles to the application of these technologies in Construction Site Safety Management (CSSM). Accordingly, the aim of this research was to identify and analyze the barriers impeding the use of such technologies in the CSSM context. To this end, initially, the extant literature was reviewed extensively and nine experts were interviewed, which led to the identification of 18 barriers. Then, the fuzzy Delphi method (FDM) was used to calculate the importance weights of the identified barriers and prioritize them through the lenses of competent experts in Hong Kong. Following this, the findings were validated using semi-structured interviews. The findings showed that the barriers related to “productivity reduction due to wearable sensors”, “the need for technical training”, and “the need for continuous monitoring” were the most significant, while “limitations on hardware and software and lack of standardization in efforts,” “the need for proper light for smooth functionality”, and “safety hazards” were the least important barriers. The obtained findings not only give new insight to academics, but also provide practical guidelines for the stakeholders at the forefront by enabling them to focus on the key barriers to the implementation of IoT-based technologies in CSSM.

## 1. Introduction

Construction safety is considered a critical issue in every construction project due to the high number of reported fatalities and worker injuries [1]. According to the available data, there are more than 60,000 fatalities yearly in construction projects around the globe [2]; the construction sector is responsible for 20% of the total fatalities in Europe, with this figure being much higher in developing countries at 20–40% [3]. These values are significant even when it comes to developed countries (e.g., Hong Kong) using high-tech technologies. According to Shafique and Rafique [4], in Hong Kong, 75% of worker fatalities are related to the construction industry. These statistics show the necessity to conduct more research on various safety aspects of construction projects to minimize the fatality and injury rates.

Given the fast progress in the development of digital technologies, their adoption in construction projects has increased due to the multiple benefits promising to improve the safety environment of construction sites. According to Luo et al. [5], there is an increasing trend in conducting research in the field of digital technologies to improve the safety of construction projects, in which virtual reality, augmented reality, mixed reality, digital twins, and Internet of Things (IoT) are listed as the most useful ones. Among these technologies, IoT is capable of automating safety monitoring of construction sites and hazard detection [6]. Moreover, to take full advantage of using digital technologies in construction projects, the devices need be connected so that all data can be transferred and analyzed by data analysts and experts. IoT technology is a suitable solution for facilitating such data transmission among devices; however, similar to other new technologies, IoT is not currently considered a common practical technology in every construction site.

Although the benefits of adopting IoT-based technologies for various aspects of Construction Site Safety Management (CSSM) have been discussed in the literature, the adoption of these technologies is in its infancy stage, even in developed countries such as Hong Kong. There is a lack of research identifying and analyzing the barriers to the further adoption of IoT-based technologies for improving occupational health and safety (OHS) within the construction sector. Therefore, this study aims to achieve the following objectives:

(1) To identify the barriers to applying IoT-based technologies to CSSM;
(2) To prioritize the identified barriers using a fuzzy-based algorithm.

The novelty of this research is the investigation of the barriers associated with the implementation of leading-edge technologies in the CSSM context within developed countries. The remainder of the paper is structured as follows: Section 2 reviews the literature pertaining to the use of IoT-based technologies in CSSM. Section 3 explains the details of the research methodology in achieving the research objectives. Section 4 shows the results and discusses the research findings. Finally, the concluding remarks of the study, the implications of the findings, the limitations, and the directions for future work are presented in Section 5.

## 2. Literature Review

In recent times, the dynamic evolution of the construction industry has been witnessed for meeting modern-day infrastructure development challenges. To tackle these challenges, not only construction site safety, but also other critical aspects such as time, cost, and quality need to be well managed. Safety is of the utmost importance in many sectors, and the construction sector is no different. Moreover, the application of innovative concepts, tools, and theories is key to the solution of the current safety challenges. In this context, this paper investigates the adoption of IoT-based technologies in the construction site safety domain and attempts to explore the existing barriers. In the following paragraphs, the recent literature on the topic defined above is reviewed in detail. 

Zhou et al. [7] used IoT technologies (i.e., radio frequency identification (RFID), ultrasonic detection, and infrared access technologies) through a three-tier network architecture to develop a safety barrier warning system for underground construction sites. The proposed system was implemented in the underground construction of the Yangtze River crossing project to generate early warnings and alarms for the hazards at the site. It showed improvements in the safety performance by reducing the number of accidents. 

Antwi-Afari et al. [8] examined the application of IoT to ergonomic risk assessment by identifying the awkward working postures of construction workers using a wearable insole pressure system. Awkward working postures lead to non-fatal occupational injuries to workers, which, in turn, result in poor construction productivity, hence affecting the overall project performance in a negative manner. The authors believed that there is great potential for the application of their proposed wearable insole pressure system, as it has both practical uses and economic benefits due to the usage of IoT technologies including sensors, vision-based technologies, and wireless communication. Sigcha et al. [9] believed that wearable technology, such as microelectronic mechanical systems (MEMS), accelerometers, and smartwatches had witnessed tremendous evolution in the domain of safety research. Among these, smartwatches are easy to use and have broad applicability, e.g., integrating an accelerometer with a smartwatch for precise motion detection, and considering any uncertainty effects in the occupational risk assessment. 

Soltanmohammadlou et al. [10] addressed the construction site safety issue by providing an in-depth review on the real-time locating systems (RTLS) used for better site safety. It was observed that RTLS could facilitate the safety management process in various pertinent research directions, such as accident prevention, safety monitoring, safety alerts and warnings, behavior-based safety, physiological status monitoring, communication-based safety, ergonomics analysis, and on-site safety trainings. Moreover, the following IoT technologies were identified in their review: locating sensors, vision-based technologies, ultra-wide band technologies, Bluetooth, Zigbee, ultrasound, and infrared technologies. Costin et al. [11] identified real-time feedback, global positioning systems (GPSs), lasers, geographic information systems (GIS), accelerometers, gyroscope sensors, RFID technology, Bluetooth systems, and wearable sensors as being considered IoT technologies. Based on that, they proposed a conceptual IoT-based framework to generate active leading indicators (ALIs) that had the potential to identify safety hazards and prompt immediate actions to prevent incidents. The use of IoT assists the collection of quantifiable data and triggers an actionable response in real-time based on defined thresholds. In addition, the interactive physical–virtual feedback loop is a vital component of the proposed IoT system. The case study findings validated the IoT-based ALI framework and demonstrated the feasibility of the system. 

In another study, Zhang et al. [12] used smart phones and sensors such as accelerometers and gyroscopes as tools to find and realize construction workers’ near miss falls based on the artificial neural network. A loss of balance situation was created using a balance board to simulate the near miss fall event in the training and evaluation phases of the experimentations. The smartphones are used for data acquisition purposes on sites with an average error-detection rate of 16.26%; thus, if adopted on a large scale, these devices can be useful in improving site safety. Zhou et al. [13] utilized RFID-based location and tracking technology, ultrasonic detection technology, and infrared access technology to propose a cyber–physical system-based safety monitoring system for blind hoisting in metro and underground construction projects. The proposed IoT-based system, through simulating and monitoring, helps to prevent accidents that occur in the dynamic hoisting process. Moreover, the results of a case study showed that the proposed system could be effectively applied to several cases, for example, dams, high-rise buildings, and large infrastructure projects. 

Chung et al. [14] presented an IoT-based application for monitoring construction site safety in the Hong Kong construction sector. The study first investigated the effectiveness of mandatory basic safety training delivered to construction workers. Afterwards, an IoT-based innovative safety model was designed to provide real-time monitoring of the construction site personnel and environment. In the end, a cost comparison was provided, which suggested significant cost savings with respect to the traditional manual systems. Okpala et al. [15] assessed the feasibility of integrating IoT into safety management systems (SMS), with specific focus on wearable sensing devices (WSDs) and location tracking biosensors. A structured questionnaire was used to assess the usefulness of WSDs in the SMS context. In addition to the increased utility of WSDs, the analysis revealed that the use of WSDs provides useful information on the safety and health of workers, offers value for money, has the potential to prevent accidents and its associated costs, and provides compatible and seamless solutions to the concerned stakeholders. Furthermore, interoperability and standardization were found to be key challenges. Asadzadeh et al. [16] presented a comprehensive systematic review of the existing literature on the use of IoT sensors in the safety management context for the construction industry. The findings reflected a strong inclination of the researchers working in this field to adopt IoT technology, such as sensor-based technologies, accelerometers, and gyroscopes, to solve safety issues due to the unsafe and hazardous nature of the construction industry. 

Furthermore, the integration of different sensor-driven systems with information modeling technologies such as BIM for the improvement of construction safety has also been addressed in the recent literature. Yang et al. [17] utilized IoT technologies including Wi-Fi modules, photoresistors, optical sensors, force stretchable resistors, and touch sensors for productivity improvement by developing an automated personal protection equipment (PPE)-tool pair checking system using IoT with Wi-Fi modules attached to the PPE. The developed automated system simultaneously warns the user and the safety officer about the improper usage of PPE. The authors’ belief about the system being efficient and effective in relation to the productivity and site safety improvement was well supported by the detailed lab experiments as well. With the rise in the usage of IoT and its accompanying technologies, Häikiö et al. [18] investigated construction workers’ attitudes using an online survey towards the acceptability of IoT-based wearable technologies. Based on the analysis of over 4000 survey forms, it was found out that privacy and security related to the wearables in the workplace were the main concerns of the workers. User acceptance and trust building are known to be crucial aspects for better adoption.

Regarding the implementation of IoT into the construction safety domain, Rey-Merchán et al. [19] proposed a virtual fence system based on Bluetooth Low Energy (BLE) beacon technology to avoid the intrusion of workers into hazardous areas. The designed system was then evaluated by a structured questionnaire distributed to the industry experts. The findings indicated that the system was not only inexpensive, but also convenient to integrate and configure; some other factors, such as top management support, social acceptance, alignment with organizational culture, and legislations, will also boost its applicability. Ghosh et al. [20] are of the opinion that sensor-based integrated virtual IoT technologies provide exciting opportunities for the construction sector to solve a range of problems, and with the use of a scientific mapping tool, different patterns and trends of IoT research in the construction sector have been explored as well. Having reviewed the corpus of literature, it was found that there is still a limited number of studies undertaken in this field. Huang et al. [21] considered augmented hearing protection technology in addition to the explored critical drivers of IoT adoption, which includes interoperability, data privacy and security, flexible governance structures, and proper business planning and models.

From a review of the potentials of IoT technology adoption in the construction site safety domain (as shown in Table 1), it was well observed that most of the studies were quite recent (i.e., undertaken in the last 2–3 years) and did not focus much on the documentation and detailed evaluation of the barriers to the extensive adoption of IoT-based technologies, such as RFID, ultrasonic detection and infrared access technologies, sensors, vision-based technologies, wireless communication, wearable technologies, MEMS, accelerometers, smart devices and watches, location sensors, ultra-wide band (UWB) technologies, Bluetooth, Zigbee, real-time feedback, GPS, lasers, GIS, gyroscope sensors, location tracking, biosensors, Wi-Fi modules, photoresistors, optical sensors, force stretchable resistors, touch sensors, BLE beacons, integrated physical and virtual technology, and augmented hearing protection technology. Furthermore, the mentioned studies in Table 1 merely discussed the generic barriers to the adoption of such technologies. Therefore, the literature suffers from a clear gap that needs to be filled, which constitutes the scope of this study. 

## 3. Methodology

**Figure 1:**
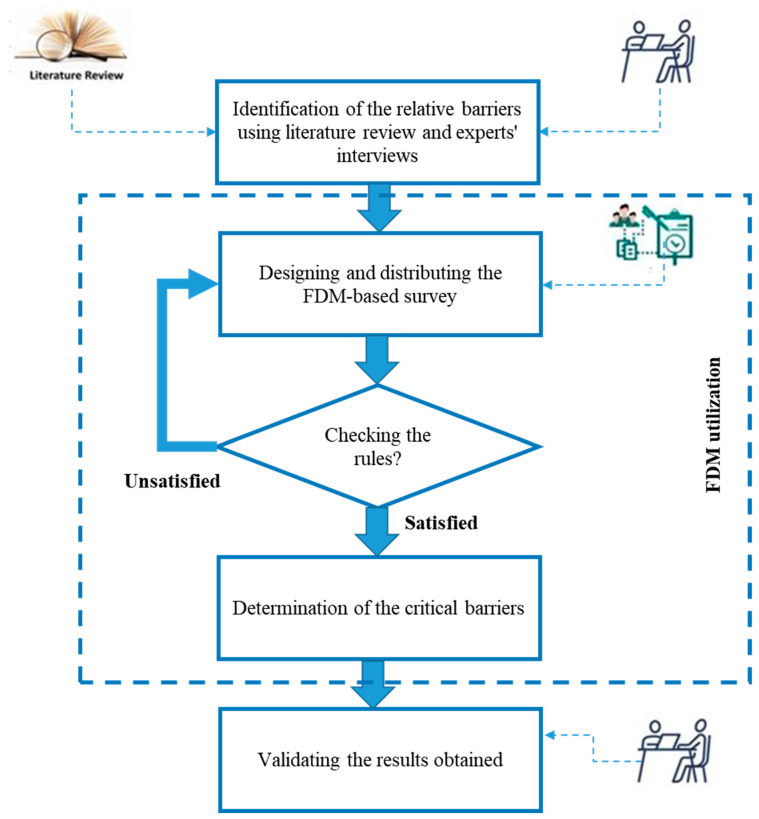
Research methodology taken for determination and ranking of barriers to IoT-based technologies implementation in CSSM.

In this section, the methodology adopted for identifying and analyzing the barriers to the implementation of IoT-based technologies in CSSM is explained in detail. Figure 1 illustrates the different steps undertaken towards achieving the objectives specified for this research. First, a comprehensive literature review for the identification of the relative barriers was carried out, after which the list of identified barriers was presented to nine experts who were asked to add any missing items from their viewpoints. Then, to investigate the importance and prioritize the identified barriers, the fuzzy Delphi method (FDM) was employed. Finally, to validate the results, several interviews were held with the experts. The steps involved in the methodology of the current paper are illustrated in Figure 1.

### 3.1. Data Collection

In order to obtain practical results, the researchers attempted to select qualified experts with relevant experience (in the context of Hong Kong) and knowledge in this domain. To this end, various criteria (as suggested in [23,24]) were considered in the selection of qualified panels. The criteria used in this study were: (1) to have at least an undergraduate degree relevant to the area, such as in construction engineering, construction management, architecture, or building (this criterion was taken into account to ensure that the respondents were aware of the technical aspects of building projects); and (2) to have at least five years of relevant experience with construction safety; they should be involved in checking and investigating the safety operations being undertaken on site (as managers, engineers, supervisors, or operators in the daily construction activities). Notably, to select the qualified experts for satisfying the second criterion, only those who had five years of experience together with the experience of working in a reflective project (where any IoT-based technologies were adopted) were considered. The second criterion was defined in order to make sure that the respondents had first-hand experience of the technical aspects targeted in this research. The two above-mentioned criteria led to the selection of nine experts for FDM, and five for the validation stage (as shown in Table 2).

### 3.2. Identification of Barriers

To obtain an exhaustive list of barriers to the adoption of IoT-based technologies in CSSM, a two-step approach was undertaken. First, a comprehensive literature review on the publications published on the topic was conducted, which led to the identification of 18 barriers. Then, an online interview with the senior experts (described in the previous section) was carried out. The list of identified barriers was sent to the selected senior experts and, accordingly, they were asked to add any items missing from the list. Notably, in the interviews undertaken to add more barriers to the list presented to the experts, all of them unanimously stated that the prepared list was quite exhaustive; thus, no more barriers were added.

### 3.3. Prioritization of the Identified Barriers Using the Fuzzy Delphi Method

The literature consists of many studies that have adopted the Delphi method in order to elicit, refine, and draw upon the collective opinions of a number of experts about a specific subject [25]. Many scholars use this method with the aim of alleviating the adverse impacts of group interactions and providing equal opportunities to all people participating in a study to share their viewpoints and take part in decision making processes [26]. The Delphi method collects all participating experts’ opinions using anonymous questionnaires; afterwards, when the answers are exposed to statistical analyses, the final results are fed back to the experts to be modified again if required. Finally, researchers who make use of this method expect to obtain convergence in the experts’ opinions [27]. On the other hand, several studies have mentioned that the drawback of the traditional Delphi method is the low convergence of the experts’ opinions, as well as the inefficient process of the method when it is taken into action. This is because iterative inquiries are needed to achieve consensus in the experts’ opinions [23]. Furthermore, in the Delphi approach, the participants express their opinions in a verbal manner. This is a challenge, given the fact that verbal expression cannot fully reflect an individual’s real thinking styles and it often fails to show their mental latencies. Therefore, a fuzzy set theory (FST) was proposed with the aim of effectively addressing the issues in relation to the subjectivity, ambiguity, and fuzziness of people’s judgments. FST was able to quantify the linguistic facets of the available data and the preferences for group or individual decision-making sessions [28]. FST is, in fact, a developed version of the traditional set theory, where the elements of a set possess the membership grades ranging between 0 (non-membership) to 1 (full membership) [29]. With this in mind, the following steps were involved in the execution of the FDM for ranking the identified barriers and determining those considered most critical. 

**Step 1.** Designing the FDM-based questionnaire survey. After determining the obstacles to the implementation of IoT in CSSM, a structured questionnaire was provided on the basis of the identified barriers, using the linguistic variables presented in Table 3 (starting from very low importance to very high importance), as suggested in [30]. Afterwards, nine experts were invited to fill out the questionnaire with the use of the defined linguistic variables. Notably, due to the fact that triangular fuzzy sets are considered in this study, each fuzzy set is comprised of three values, namely the lowest possible value, the most likely values, and the highest possible value. As can be seen, the bounds (the lower and the upper) are considered within the range of 1 and 5, while the membership functions are within the range of 0 and 1. A sample of the questionnaire is provided as Appendix A. It is worth mentioning that based on the qualifications of the experts participating in this research, all were fully aware of the challenges associated with the adoption of IoT-based technologies with regard to CSSM.

**Step 2.** Checking the consensus of the responses provided. Once the questionnaires had been filled out by the experts, then there was a need to check whether the consensus among the pool of experts had been reached or not. To this end, the following two rules were considered in this study, as proposed in [31]:

(a) If the standard deviation to mean ratio (SDMR) for each barrier is less than 30%, then a good level of consensus among the pool of experts for that specific barrier is deemed reached. On the other hand, if the SDMR for each barrier is equal to or more than 30%, then the level of consensus among the pool of experts for that specific barrier is considered poor. Accordingly, the respective experts need to adjust their responses. It is notable that the SDMR needs to be calculated for each barrier separately, based on all of the questionnaires filled out by the pool of experts.
(b) If the Cronbach reliability test corresponding to the responses of an expert is less than 0.7, then the answers provided are not prudent and, consequently, need to be done again; otherwise, the provided responses are concluded to be sagacious and consistent. It is worth mentioning that the Cronbach reliability test needs to be calculated for checking the consistency of the responses of an expert in filling out the respective survey.

**Step 3.** Determining the critical barriers. After reaching consensus among the experts, then the answers provided needed to be quantified. The used FDM makes use of triangular fuzzy numbers (TFNs) to retain the key barriers so as to quantify the variables assigned to the identified barriers. Therefore, the max and min values of the experts’ opinions are considered the two terminal points of the TFNs. The arithmetic mean is considered the membership degree of the TFNs when deriving the statistical unbiased impact. In addition, it helps evade the effects of extreme values. For that reason, the use of TFNs brings simplicity, since it covers the opinions of all of the participating experts in a single investigation [2]. After the questionnaire was filled out by the experts, the linguistic variables allocated to each barrier were quantified by the research team. To this end, Equations (1) and (2) were utilized for the calculation of the aggregation of the experts’ feedback for g barriers:

(1) $$F_{i}\left(b\right)=\left(l_{b},m_{b},u_{b}\right),\;\;for\;i=1,2,\ldots ,n\;$$

(2) $$B\;\left(b\right)=\left(l_{B},\;m_{B},\;u_{B}\right)=\left(min\;l_{b},\;mean\;m_{b},\;max\;u_{b}\right)$$

where $F_{i}\left(b\right)$ stands for the TFN response of expert i for barrier b, while B (b) denotes the aggregation of the responses of all of the experts for barrier b (in which $min\;l_{b},\;mean\;m_{b},\;max\;u_{b}$ represent the minimum lower bound value allocated by the experts, the mean of the most likely value allocated by the experts based on the arithmetic mean, and the maximum upper bound value allocated by the experts, respectively). Following this, the responses aggregation was subjected to the defuzzification process for the purpose of achieving a crisp value as the significance of each barrier (Equation (3)). To choose the significant barriers, there is a need to calculate a threshold value (Equation (4)) as suggested in [32]:

(3) $$D_{B\left(b\right)}=\frac{l_{B}+(4*m_{B})+u_{B}}{6}$$

(4) $$TS=\frac{\sum_{i=1}^{g}D_{B\left(b\right)}}{g}$$

where $D_{B\left(b\right)}$ stands for the defuzzified number of the aggregated responses for barrier b, and TS signifies the threshold value. If the defuzzuifed value of a particular barrier exceeds the specified threshold value, then the barrier will be chosen as a critical one; otherwise, it will be considered non-critical. Notably, for the sake of prioritization, the higher the final weights of barriers (which is denoted as $D_{B\left(b\right)}$), the more critical the respective barrier.

### 3.4. Validation Stage

To gauge the external validity of the results obtained, further interviews were held with five experts, who were not in the list of the nine experts formerly selected. To this end, semi-structured interviews were held with five qualified experts for the purpose of validating the obtained results, as suggested in the literature (e.g., [30,33]). These experts were expected to check the applicability of the findings to a bigger picture by giving their opinions in regard to the identified barriers presented to them through some open discussions. Notably, some of the interviews were carried out orally, and others using a questionnaire. Moreover, these five experts were invited to rate the significance of the identified barriers with the help of a five-point Likert scale. The purpose of this validation was to check the “external validity”—which refers to the generalization of the findings.

## 4. Results and Discussion

**Figure 2:**
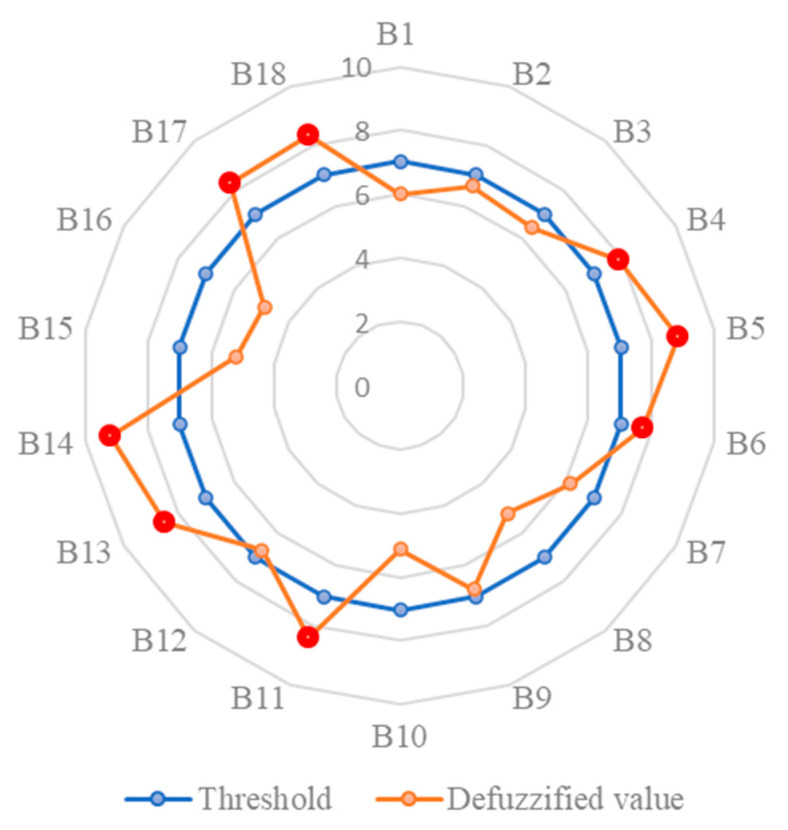
Critical barriers based on the specified threshold values.

In this research, the barriers to the adoption of such technologies were investigated in the Hong Kong context. For the sake of brevity, only the gist of the results is provided hereinafter. Table 4 illustrates the results obtained with the use of FDM. As can be seen in this table, the consensus was reached in the second round of the surveys, considering the two conditions specified for the used FDM (conditions (a) and (b) stated in Step 2); SDMR for five barriers crossed 30%; therefore, the corresponding surveys containing these barriers were sent to the respective experts to adjust their ratings). Notably, in the second survey, an acceptable level of reliability among the pool of experts was achieved since the proposed reliability tests exceeded 0.7. To make it more explicit, the relative tests for all of the experts were above 0.7, and the aggregation reliability tests, which were based on the aggregation of the Cronbach tests for all experts, were equivalent to 0.7884. It can be observed that eight barriers were perceived to be critical to the adoption of IoT-based technologies in CSSM in Hong Kong. This shows the fact that although the adoption of these technologies has brought about tangible benefits, there are some barriers that might act as stumbling block to the further utilization of such technologies. These critical barriers are B4, B5, B6, B11, B13, B14, B17, and B18, as can be seen in Figure 2. 

According to the findings (Figure 2), B14 (i.e., “productivity reduction due to wearable sensors”) is the most important barrier hindering the adoption of IoT in the Hong Kong construction industry, followed by B5 (“the need for technical training”) and B13 (“the need for continuous monitoring”). This shows the difficulties that the construction workers face by wearing such sensors, which could reduce their productivity. This finding indicates that although the findings of previous research (e.g., [9]) show the considerable improvements made in using hardware, much more effort is needed to develop more user-friendly sensors (hardware) to be attached to workers. In addition, the second and third most important barriers indicate the significance of the technological aspects of using IoT from the workers’/supervisors’ perspectives towards the monitoring and maintenance of devices. It can be stated that the higher the knowledge of construction stakeholders regarding every aspect of adopting IoT-based technologies in construction projects, the higher the chance of its employment and the safer the construction sites.

Apart from the above most significant barriers, the findings showed that the high importance of B11 (“data privacy issues”), B17 (“low reliance on the technology”), and B18 (“poor governmental policies and incentives”) cannot be ignored. In terms of B11, the findings of this research are consistent with those reported by the authors in [18], indicating the reluctance of workers to use IoT-based wearables. This barrier could be overcome by managers through familiarizing the workers with the importance of collecting such data from their activities, and building trust among construction managers and workers. When it comes to B17, the findings revealed that despite numerous practical advancements and innovations in developing and adopting technologies, there has been a lack of trust regarding the use of these new technologies among workers/managers. This could be addressed by providing relevant training and statistics from the government to construction managers and from managers to the workers—as discussed in the literature in detail [1]—to show the effectiveness of adopting technology in construction safety management. Due to the lack of appropriate regulations and guidelines stipulated by the government, the adoption of aforesaid technologies has not been fully achieved yet in Hong Kong. In this regard, all of the interviews affirmed that there is a need to introduce some incentives and make new policies upon the utilization of these technologies, such as long-term loans for contractors, promotion or bonus points for the site supervisors responsible for checking the safety of construction-related tasks, or tax deductions for owners/clients. Such considerations might alleviate the status quo of the mentioned barriers, paving the way towards automating construction site safety management. 

On the other hand, the least significant barriers, i.e., B16 (“limitations on hardware and software and lack of standardization in efforts”), B10 (“the need for proper light for smooth functionality”), and B15 (“safety hazards”) indicate that Hong Kong, as a developed Asian country, is ready for the wide adoption of IoT in construction projects, in terms of providing hardware, software, and standards of use. Given the significance of B14 and the insignificance of B16, it can be understood that if the technology used in wearable sensors is improved, the adequacy of the hardware and software and the standards of using them would be enhanced in Hong Kong. This finding could be applicable to developed countries, while according to Tabatabaee et al. [34], many construction companies in developing countries are experiencing the lack of both hardware and software, even when using older technologies such as building information modelling. Consequently, the significance of B16 should not be overlooked when it comes to developing countries.

In terms of B15 as a much less significant barrier, it can be understood that construction workers are faced with no additional safety hazards/accidents when using IoT devices. This is due to the fact that the adoption of IoT-based technologies (e.g., smart wearable sensors) prevents the workers from being involved in perilous activities on construction sites when the respective hazard is detected in advance, which is in line with the findings in the literature (e.g., [11]). That is to say, sensors mounted on the worker’s body act as a deterrent to their involvement in the detected hazardous zones; thus, there would be a very slight chance of the worker ignoring the occurrence of a hazard or an accident.

After obtaining the results, further interviews were conducted with senior experts for checking the external validity of the findings. Table 5 illustrates the gist of the interviews. As can be observed, there is an acceptable level of consistency between the rankings obtained in the main round of study and those of the validation stage. Taking the most critical barriers as examples, B5, B14, and B13 are seen as the most crucial barriers to the further adoption of IoT-based technologies for site safety monitoring. This finding, which was obtained from the experts, is in line with the main results of this study; accordingly, the findings of this study are of good external validity, although the rankings of a few barriers might be different in both cases. For instance, there are some barriers placed in the same spot, which is due to the employment of traditional Likert-scale questioning. In other words, another interesting observation during the validation stage was that the use of FDM for quantifying the importance of the identified barriers led to obtaining more diversified rankings against those of the common Likert-scale approach. Such diversification in terms of the final rankings of the barriers is intertwined with the utilization of fuzzy sets during both the fuzzification and defuzzification stages.

## 5. Conclusions and Recommendations

A considerable number of fatalities and injuries are reported in construction projects annually, and the adoption of digital technologies could provide safer construction sites. The adoption of IoT-based technologies in construction projects has offered myriad benefits, especially in the field of construction safety management. However, its adoption is in the early stage, even in developed countries. This paper reviewed the existing literature regarding the barriers to the adoption of IoT-based technologies in construction site safety management (CSSM). Then, the most significant barriers were determined using a comprehensive fuzzy Delphi method. Once the external validity of the findings had been checked through semi-structured interviews, 18 barriers were found to hamper the adoption of IoT-based technologies in CSSM. Based on the outcome of the external validity, it was concluded that the findings of this research have high potential for generalization in the context of Hong Kong. The novelty of this research is twofold: first, the identification of obstacles to the use of IoT-based technologies in the safety management context, and second, prioritizing the identified obstacles in a developed country—which is, to the best of the authors’ knowledge, the first of its kind. The findings of this research give new insight to academics in the field of safety management; they can be used either for further investigations, or for developing conceptual frameworks. In addition, construction safety managers may use the findings of this research to improve the safety of construction sites by overcoming the most critical barriers to the use of IoT-based technologies.

The findings of this study are built upon the opinions of experts within the context of Hong Kong, and could be considered a snapshot of the barriers to the use of IoT-based technologies in safety management, although these are not futureproofed. In addition, it is worth mentioning that the identified barriers might be merely due to the implementation costs rather than technical and safety perspectives. Given the differences between the financial, technological, and technical aspects among countries, and also the fast pace of technological advancements, the identified barriers should be reinvestigated to be used in other research or by safety managers. Future research is needed to investigate the relationships among the barriers identified in this study. Moreover, the barriers listed in this research could be separately reinvestigated with respect to different technologies used for IoT. In addition, there is still a lack of research on the application of IoT in safety management, and other aspects of IoT adoption, such as strengths, weaknesses, opportunities, and risks, need to be analyzed. On top of all this, it is noteworthy that this study only focuses on the corresponding sub-categories to make the application of the used methodological approach more reproducible for concerned parties. In this way, they can draw their attention to controlling the existing sub-barriers (rather than focusing on the main barriers as well) in the first place. However, another potential future approach may be to couple the importance of the main and sub-barriers in a single study, and accordingly, make comparisons of the results with those of this study.

## Figures and Tables

**Table 1:** List of barriers to the adoption of IoT.

Barrier	Description	Reference
Lack of integration between technologies (B1)	Solutions that combine different technologies (e.g., BIM) with vision-based monitoring systems have not been sufficiently explored.	[9,16,22]
Limited scale of technology implementation (B2)	Most of these technologies tested their proposed algorithms on datasets that are proprietary to specific projects, which are bound by specific project constraints.	[7,16,22]
Lack of publicly available large datasets (B3)	Lack of publicly available large datasets for construction safety monitoring causes difficulties when comparing the performance of various algorithms.	[16]
Deficiencies in onsite data recording (B4)	Data, e.g., in the form of photos taken by workers on site, are mostly unorganized and stored locally.	[7,14,15,16]
The need for technical training (B5)	Proper technical training for workers and owner involvement are essential to prudently work with sensors functionalities (both workers and supervisors).	[15,16]
The need for high computational efficiency (B6)	For smooth functionality and effective data synchronization, high computational efficiency is critical.	[17]
The need for heavy batteries (B7)	Wi-Fi module is not an energy-saving solution for such technologies, thus requires high capacity and heavyweight batteries.	[11,17]
False alarms (B8)	Due to technological glitches and device registration issues, false alarms are quite common.	[7]
The need for off-line sensor network (B9)	For situations such as underground construction sites or isolated construction sites, neither the Wi-Fi nor general packet radio service (GPRS) are available, and the system would fail to upload data and receive orders. To address the communication coverage issue, an off-line sensor network is needed.	[7,10,17]
The need for proper light for smooth functionality (B10)	Systems might stop working or fail to detect wearing motions due to the constantly low illumination.	[17]
Data privacy issues (B11)	Workers are hesitant to adopt technology due to identity disclosure and related data privacy issues.	[7,18,19]
Challenges arising from physical interactions (B12)	Due to the wearable technological gadgets and involvement of high-tech solutions, physical interaction between workers is quite challenging.	[7]
The need for continuous monitoring (B13)	To achieve enhanced durability of the technological advancements, continuous monitoring and debugging of devices are essential.	[7,15]
Productivity reduction due to wearable sensors (B14)	Such technological methods require sensors to be attached to the workers’ skin, which makes them feel uncomfortable and is inconvenient when performing a given task, eventually reducing productivity.	[8]
Safety hazards (B15)	Workers may exhibit high-risk behavior by ignoring prompts from the devices.	[15]
Limitations on hardware and software and lack of standardization in efforts (B16)	Since the field of study is emerging, there is still a lack of standardization efforts; therefore, there are limitations on both hardware and software.	[11,15]
Low reliance on the technology (B17)	Due to fear of the unknown and lack of concrete examples, users have low reliance on the technology and still believe in ‘old school’ solutions.	[15,18,21]
Poor governmental policies and incentives (B18)	Despite governments having invested significantly in research and development for technological advancements, the policies and related incentives have not been well defined, thereby resulting in low adoption.	[21]

**Table 2:** Profile of experts involved in the study.

Experts’ ID	Educational Level	Title	Experience (Year)	Experience (Year) in Using IoT-Based Technologies	FDM Stage	Validation Stage
1	Bachelor’s in civil engineering	Contractor	14	6	*	—
2		Site supervisor	6	3	*	—
3		Contractor	22	3	*	—
4		Project manager	25	5	*	—
5		OHS officer	22	2	*	—
6		Site supervisor	10	2	*	—
7	Master’s in construction management	Project manager	14	4	*	—
8		OHS officer	16	4	*	—
9	Master’s in building services	Site supervisor	17	4	*	—
10	Bachelor’s in civil engineering	Contractor	19	3	—	*
11		Site supervisor	14	5	—	*
12	Master’s in construction management	Safety manager	12	2	—	*
13		Facility manager	13	5	—	*
14		Project manager	18	2	—	*

Note: * denotes the involvement of the respective expert at the stage, while — denotes that the respective expert is not involved at that stage.

**Table 3:** Linguistic variables used for determining the importance of the barriers towards the adoption of IoT in CSSM.

Variables	Fuzzy Numbers
Very low importance	(1,1,1.5)
Low importance	(1.5,2,2.5)
Medium importance	(2.5,3,3.5)
High importance	(3.5,4,4.5)
Very high importance	(4.5,5,5)

**Table 4:** Results of FDM (for the first and second rounds).

Barrier	Min	Most Likely Value	Max	Defuzzification	Rank	SDMR % (1st Round)	SDMR % (2nd Round)
B1	1.50	3.0000	4.50	6.0000	14	35	18
B2	1.50	3.3684	5.00	6.6579	11	24	24
B3	1.50	3.2105	5.00	6.4474	12	18	18
B4	2.50	4.0526	5.00	7.9035	7	7	7
B5	3.50	4.5263	5.00	8.8684	2	9	9
B6	2.50	3.9474	5.00	7.7632	8	11	11
B7	1.50	3.0000	5.00	6.1667	13	26	26
B8	1.50	2.4211	4.50	5.2281	15	33	24
B9	1.50	3.4737	5.00	6.7982	9	37	16
B10	1.00	2.4737	4.50	5.1316	17	25	25
B11	2.50	4.4211	5.00	8.3947	4	13	13
B12	1.50	3.4211	5.00	6.7281	10	15	15
B13	2.50	4.5263	5.00	8.5351	3	26	26
B14	3.50	4.7895	5.00	9.2193	1	39	24
B15	1.00	2.5263	4.50	5.2018	16	33	16
B16	1.00	2.3158	4.50	4.9211	18	27	27
B17	2.50	4.3158	5.00	8.2544	6	13	13
B18	2.50	4.3684	5.00	8.3246	5	11	11
Agg. Reliability Tests (1st round)							0.5359
Agg. Reliability Tests (2nd round)							0.7884

**Table 5:** Rankings of the identified barriers at the validation stage against the main results.

**Barrier**	**Rank (Validation)**	**Rank (Main Results)**	**Barrier**	**Rank (Validation)**	**Rank (Main Results)**
B1	15	14	B10	16	17
B2	12	11	B11	4	4
B3	11	12	B12	10	10
B4	9	7	B13	3	3
B5	1	2	B14	1	1
B6	7	8	B15	18	16
B7	13	13	B16	16	18
B8	13	15	B17	6	6
B9	9	9	B18	4	5

## Data Availability

Data sharing not applicable.

## Appendix A

The second section of the designed FDM-based survey, which is concerned with the importance of the identified barriers, is appended as follows.

### Questionnaire Survey

Please kindly define the importance of the identified barriers to the adoption of IoT-based technologies for construction site safety management in Hong Kong. In doing so, you need to assign a linguistic variable to each barrier (in a way to best represent the importance of a particular barrier).

**Barrier**	**Importance of Barriers**				
	**Very Low**	**Low**	**Medium**	**High**	**Very High**
Lack of integration b/w technologies	☐	☐	☐	☐	☐
Limited scale technology implementation	☐	☐	☐	☐	☐
Lack of publicly available large datasets	☐	☐	☐	☐	☐
Deficiencies in onsite data recording	☐	☐	☐	☐	☐
The need for technical training	☐	☐	☐	☐	☐
The need for high computational efficiency	☐	☐	☐	☐	☐
The need for heavy batteries	☐	☐	☐	☐	☐
False alarms	☐	☐	☐	☐	☐
Off-line sensor network also needed	☐	☐	☐	☐	☐
The need for proper light for smooth functionality	☐	☐	☐	☐	☐
Data privacy issues	☐	☐	☐	☐	☐
Physical interaction is challenging	☐	☐	☐	☐	☐
The need for continuous monitoring	☐	☐	☐	☐	☐
Wearable sensors reduce productivity	☐	☐	☐	☐	☐
Safety hazards	☐	☐	☐	☐	☐
Limitations on hardware and software and lack of standardization in efforts	☐	☐	☐	☐	☐
Low reliance on the technology	☐	☐	☐	☐	☐
Poor governmental policies and incentives	☐	☐	☐	☐	☐
